# Soil inoculation with symbiotic microorganisms promotes plant growth and nutrient transporter genes expression in durum wheat

**DOI:** 10.3389/fpls.2015.00815

**Published:** 2015-10-02

**Authors:** Sergio Saia, Vito Rappa, Paolo Ruisi, Maria Rosa Abenavoli, Francesco Sunseri, Dario Giambalvo, Alfonso S. Frenda, Federico Martinelli

**Affiliations:** ^1^Dipartimento di Scienze Agrarie e Forestali, Università degli Studi di PalermoPalermo, Italy; ^2^Fondazione A. e S. Lima Mancuso, Università degli Studi di PalermoPalermo, Italy; ^3^Dipartimento di Agraria, Università Mediterranea di Reggio CalabriaReggio Calabria, Italy

**Keywords:** mediterranean, organic N uptake, plant growth promotion, Gene Expression Regulation, field experiments, arbuscular mycorrhizal fungi (AMF), plant growth-promoting rhizobacteria

## Abstract

In a field experiment conducted in a Mediterranean area of inner Sicily, durum wheat was inoculated with plant growth-promoting rhizobacteria (PGPR), with arbuscular mycorrhizal fungi (AMF), or with both to evaluate their effects on nutrient uptake, plant growth, and the expression of key transporter genes involved in nitrogen (N) and phosphorus (P) uptake. These biotic associations were studied under either low N availability (unfertilized plots) and supplying the soil with an easily mineralizable organic fertilizer. Regardless of N fertilization, at the tillering stage, inoculation with AMF alone or in combination with PGPR increased the aboveground biomass yield compared to the uninoculated control. Inoculation with PGPR enhanced the aboveground biomass yield compared to the control, but only when N fertilizer was added. At the heading stage, inoculation with all microorganisms increased the aboveground biomass and N. Inoculation with PGPR and AMF+PGPR resulted in significantly higher aboveground P compared to the control and inoculation with AMF only when organic N was applied. The role of microbe inoculation in N uptake was elucidated by the expression of nitrate transporter genes. *NRT1.1, NRT2*, and *NAR2.2* were significantly upregulated by inoculation with AMF and AMF+PGPR in the absence of organic N. A significant down-regulation of the same genes was observed when organic N was added. The ammonium (NH_4_^+^) transporter genes *AMT1.2* showed an expression pattern similar to that of the NO_3_^-^ transporters. Finally, in the absence of organic N, the transcript abundance of P transporters *Pht1* and *PT2-1* was increased by inoculation with AMF+PGPR, and inoculation with AMF upregulated *Pht2* compared to the uninoculated control. These results indicate the soil inoculation with AMF and PGPR (alone or in combination) as a valuable option for farmers to improve yield, nutrient uptake, and the sustainability of the agro-ecosystem.

## Introduction

Plants live in the soil engaging a wide range of interaction with soil microorganisms. Such interaction can include a benefit, a disadvantage or a null effect on plant growth and nutrient uptake and such an effect also depends on soil conditions, especially nutrient availability for the plant and the microorganisms.

Arbuscular mycorrhizal fungi (AMF) and plant growth-promoting rhizobacteria (PGPR) are important components of the soil microbiota and usually have major effects on plant growth under stressing conditions thanks to their ability to influence many pivotal physiological processes of both the plant, such as seed germination rate, root growth and branching, photosynthetic rates, etc., and soil, e.g., aggregate stability, pH, activity of pathogens, and so on (Berg, 2009; Venkateshwaran et al., 2013). In addition, AMF can also provide alternative nutrient uptake pathways (Finlay, 2004), which are particularly important for plant growth when nutrient availability is low. For example, the mobility of phosphate (Pi) is low, especially in alkaline soils, and its uptake rapidly leads to the development of depletion zones around the roots, which further limits P uptake (Schachtman et al., 1998). Pi acquisition in plants is ensured by members of plasma membrane Pi transporter family 1 (*Pht1*; Kobae et al., 2010), which are also involved in Pi translocation among plant cells and tissues as well as Pi remobilization from senescent to novel onset organs (Lambers et al., 2008). Homologous *Pht1* genes have been characterized in many plant species, including *Arabidopsis thaliana* (Misson et al., 2004), tomato (Daram et al., 1998), maize (Nagy et al., 2006), and wheat (Liu et al., 2013). Numerous *Pht1* members act under high-affinity systems and thus play critical roles in plant Pi uptake under Pi deprivation (Bucher, 2007). The effects of AMF on the enhancement of P uptake are well known and involve different genes encoding *Pht1* transporters (Javot et al., 2007). More recently, the differential expression of two Pi transporter genes (*Pht1;3* and *Pht1;6*) in maize root colonized by different AMF was also highlighted (Tian et al., 2013).

Unlike Pi, NO_3_^-^, the dominant N form in most agricultural soils, is highly mobile, and its uptake proceeds by at least two transport systems—a low-affinity transport system (LATS; active at NO_3_^-^ concentration >0.2 mm) and a high-affinity transport system (HATS; operating within 0–0.2 mm)—that allow plants to maximize NO_3_^-^ acquisition under low NO_3_^-^-N availability. HATS is particularly important for plant nutrition when limited or no N fertilizer is applied (Malagoli et al., 2004). In bread wheat (*Triticum aestivum*), an *NRT2.1*, an important HATS family, has been isolated and characterized, and its transcript abundance decreased in roots in response to NO_3_^-^ and NH_4_^+^ (Wang et al., 2011). Furthermore, an NAR2-like protein actively interacted with *NRT2.1* to form a functional HATS effective in NO_3_^-^ transport (Orsel et al., 2006).

Arbuscular mycorrhizal fungi root colonization positively affected nitrate uptake and allocation in tomato shoot compared to an uninoculated control, preferentially mediated by a higher expression of *NRT2.3* (Hildebrandt et al., 2002), which is also responsible for long-distance N translocation in other species (Jing et al., 2012). This mechanism was confirmed by an increased expression of four different AMF-related nitrate transporter genes in mycorrhizal *Medicago truncatula* roots (Hohnjec et al., 2005).

Unlike NO_3_^-^, NH_4_^+^ tends to be buffered by interactions with negatively charged soil particles (i.e., by the cation exchange complex). Saturable and non-saturable systems operating at low and high external NH_4_^+^ concentrations, respectively, have been characterized in several plant species (von Wittgenstein et al., 2014). The uptake of ammonium at low concentrations (i.e., under high-affinity conditions) in plant roots is mediated by AMT1-type ammonium transporters (AMTs), whose activity depends on several factors, including the plant species. For example, in *Zea mays*, such transport is most probably mediated by two rhizodermis-localized transporters (ZmAMT1;1a and ZmAMT1;3; Gu et al., 2013). In addition, in mycorrhizal *Lotus japonicus* roots, an AMT (*LjAMT2;2*) is implicated in NH_4_^+^ uptake and is upregulated by the AMF partner (Guether et al., 2009).

Such as AMF, PGPR can improve the availability of nutrients for plants through different mechanisms, including soil acidification, chelation, exchange reactions, and organic acid biosynthesis (Lugtenberg and Kamilova, 2009). The effects of PGPR on plants depend on the specific interactions between microbe and crop species. The Plant responses to the inoculation of PGPR with varying Zn-mobilizing activity varied among different wheat genotypes (Abaid-Ullah et al., 2015). Microarray studies have been conducted to gain insight into gene and pathway regulatory networks in response to inoculations of PGPR in maize and *Arabidopsis* (Fan et al., 2012). Proteomic approaches have also been used to elucidate posttranscriptional regulation mechanisms (Cheng et al., 2009). However, little information is available about the regulation mechanisms of plant gene expression mediated by the PGPR–plant interaction.

Considering the ability of both PGPR and AMF to help plants take up nutrients, they could be the most important players in shifting from conventional to sustainable land management practices. The aim of the present work was to study the N and P uptake of durum wheat grown in soil inoculated with PGPR, AMF, or both and grown under conditions of different nutrient availability. Durum wheat (*cv*. Anco Marzio) was grown in the field, and the expression of key genes involved in the uptake of nitrate, ammonium, and Pi was evaluated.

## Materials and Methods

### Farm and Field Conditions and Experimental Design

A field trial was performed in the 2011–2012 growing season at Pietranera farm (Sicily, Italy; 37°30′ N, 13°31′ E; 178 m a.s.l.) on a deep, well-structured soil classified as a Vertic Xerochrept. Soil properties (0–0.60 m layer) were as follows: 52% clay, 25% sand, pH 8.2 (1:2.5 H_2_O), 16.8 g kg^-1^ total C (Walkley–Black), 1.78 g kg^-1^ total N (Kjeldahl), 92 mg kg^-1^ available P_2_O_5_ (Olsen), 1.37 g kg^-1^ total P_2_O_5_, 35 cmol kg^-1^ cation exchange capacity, 37.2% water content at field capacity, and 19.6% at the permanent wilting point. The climate of the experimental site is semiarid Mediterranean, with a mean annual rainfall of 581 mm, mostly in autumn/winter (76%) and in spring (19%). The dry season is from May to September. The mean air temperature is 15.9°C in autumn, 9.7°C in winter, 16.5°C in spring, and 24.7°C in summer. Weather data were collected from a weather station located within 100 m of the experimental site. In the 2011–2012 growing season, total rainfall from September to March was very similar to the long-term average (513 vs. 490 mm, respectively), whereas the air temperature was 1.3°C lower than the long-term average. Soil, cropped in the previous growing season with durum wheat (*Triticum durum* Desf.), was plowed to a depth of 0.30 m in the summer and then shallowly harrowed twice to control weeds and prepare suitable seedbed conditions. No herbicides were applied.

The experimental design was a split-plot design with six replications. The main plots was N application (either fertilized or not). Subplot treatments consisted of microorganism inoculation: soil inoculated with only AMF (+AMF), inoculated with only PGPR (+PGPR), inoculated with both AMF and PGPR (+AMF+PGPR), uninoculated control (NAT). Main plots were 7.5 m × 6.0 m; each main plot was spaced 1.0 m out from the next. Along the 7.5-m side, each main plot was split in 4 subplots 1.5 m wide; within the main plot, each subplot was spaced 0.5 m out from the next to avoid cross inoculation among sub-treatments. Each 0.5-m or 1.0-m wide corridors was tilled once per month to avoid AMF and PGPR movements across plots. Fertilized plots received 80 kg N ha^-1^ as an organic fertilizer (Hydrolysed leather meal, Dermazoto N11, Organazoto Fertilizzanti S.p.A., San Miniato, Pisa, Italy), with 11% N, 0.9% P, and 40% organic C applied 1 day before sowing. Inoculation with AMF included the application of the commercial AMF inoculum (Micronised Endo Mycorrhizae, Symbio, Wormley, Surrey, Great Britain). This inoculum was composed of 5% organic material and 95% AM spores, including the following AM species: *Scutellospora calospora, Acaulospora laevis, Gigaspora margarita, Glomus aggregatum, Rhizophagus irregulare* (syn *G. intraradices*), *Funneliformis mosseae* (syn *G. mosseae*), *G. fasciculatum, G. etunicatum*, and *G. deserticola.* Total spore density in the inoculum was 25 spores g^-1^ per species. The inoculum was mixed with wheat seed at a rate of 1.55 g inoculum m^-2^ and drilled simultaneously during sowing using a batch type precision seeder.

The PGPR inoculum (*Bacillus* sp. *on bran*, Symbio) was applied to the soil at a rate of 1.55 g inoculum m^-2^ at the time of sowing. The PGPR inoculum used was composed of the following species: *Bacillus amyloliquefaciens, B. brevis, B. circulans, B. coagulans, B. firmus, B. halodenitrificans, B. laterosporus, B. licheniformis, B. megaterium, B. mycoides, B. pasteurii, B. polymyxa*, and *B. subtilis*, each at a density of 2 billion cfu g^-1^. Inoculation of +AMF+PGPR was performed by applying to the soil 1.55 g AM inoculum m^-2^ and 1.55 g PGPR inoculum m^-2^ as previously described.

Durum wheat (cv. Anco Marzio) was sown in the second half of December 2011 at a rate of 350 viable seeds m^-2^ in rows 0.18 cm apart. The experimental plot consisted of eight rows 6 m long. Weeds were controlled by hand during the experiment. At wheat tillering (on 4 April 2012, i.e., 110 days after sowing), the aboveground biomass of a subplot (six rows 0.75 m long) in the middle of the plot was harvested and weighed, and a subsample of 1 kg of fresh matter was taken and oven dried at 70°C until a constant weight. The biomass was then analyzed for total N (Kjeldahl) and P (Bertramson), the latter measured after 48 h heating at 550°C and with no addition of magnesium nitrate.

### Root Infection by AMF and Rhizoplane Colonization by Bacteria

Roots from five randomly chosen plants from each plot were sampled and three root subsamples of about 3 g each were taken. The first root subsample was immediately freeze dried in liquid N to stop metabolic activity and stored at -80°C. The second root subsample was stained with 0.05% trypan blue in lactic acid according to Phillips and Hayman (1970); root colonization by AMF was then measured with the grid intersect method according to Giovannetti and Mosse (1980). The third root sample was aseptically separated from the shoots, cleared from soil with sterile forceps and saved at -80°C for further analysis. Rhizoplane bacteria were extracted according to Pereira et al. (2011). Briefly, bacteria from each root sample were submerged in sterile phosphate buffered saline (PBS: NaCl 8 g, KCl 0.2 g, Na_2_HPO_4_ 1.15 g, KH_2_PO_4_ 0.2 g, deionized water 1000 ml, pH 7.3) and serially diluted up to 10^-9^. Aliquots of 0.2 ml were taken and plated in duplicate on nutrient agar (OXOID, Milan, Italy) treated with 15 mg/l nystatin to impair fungi growth. Plates were aerobically incubated at 30°C. Colony-forming units were counted after 2, 4, and 7 days to allow for the development of slower growing colonies.

### Gene Expression Analysis

Real-time Sybr PCR analysis was performed to evaluate the expression of durum wheat genes in response to microorganism inoculation and N fertilization. The gene expression analyses were conducted at anthesis. Three biological replicates were considered for each of the eight conditions. Each replicate was a pool of 10 roots from two plants per plot. For each target gene, PCR primers were designed based on *Triticum aestivum* sequences present in NCBI (**Table 1**) and used with Sybr Mix reactions (Bio-Rad, Hercules, CA, USA). RNA was extracted from roots using the Plant Mini Extraction Kit (Qiagen, Hilden, Germany). DNase treatment and cDNA synthesis were performed in a combined protocol following the Quantitect Reverse Transcription Kit (Qiagen) instructions. A standard curve to determine the linearity of amplicon quantity versus initial cDNA quantity was generated for each gene. Amplifications using 25 ng cDNA in a 15-μL final volume were performed. Amplifications were conducted on a Bio-Rad iQ5 PCR system using standard amplification conditions: 10 min at 95°C, 40 cycles of 15 s at 95°C, and 1 min at 60°C. All PCR reactions were performed in duplicate. The qPCR results were analyzed using the 2^-ΔCt^ comparative method as previously described in the Real-Time PCR Application Guide (Bio-Rad) and by Livak and Schmittgen (2001). Based on the fluorescence logarithmic graph, the appropriate threshold was chosen and the Ct was measured with an autocalculated threshold following baseline subtraction. The *18S* gene of *Triticum aestivum* (AB778770) was shown to have more constant expression than *elongation factor 1*. Thus, *18S* was used as a reference gene. Relative changes in expression were determined by calculating the ΔCt between the target (Ct sample) and reference (Ct 18S) genes.

**Table 1:** List of primers used in qRT-PCR analysis.

Gene		Primer sequences
Pht2	AJ344242	F: TTGGAGGAGTTGTACCGCAT R: TAGAGCACGACGAAACCAGT R: AGGCAGGAGACAGGTGAAAA
PT2-1	AY293827.1	F: TACATGCAGGTCCTGTCAGC R: TATCTCAGCGCTGCTTGCTA
Pht1	AJ830009.1	F: TGATCATGGGCTCCTTCCTC R: ACCAGGTGACAATGCAACC
NRT1.1	AY587265.1	F: CACAGCGAATAGGGATTGGT R: CGCCTAGCAGGAAGTACTGG
NRT2	AF288688.1	F: GTGGTGCCACACAACTCATC R: TTCTGGAGACTCGCAAGGTT
NAR2.2	AY763795.1	F: CCTCTCCAAGCTTCCTGTGA R: CGTAGCAGAGGCTGACCTT
AMT2.1	AY428038.1	F: AGCCGAACCTCTGCAATCTA R: TGACGACGCAGATAATGGAC
AMT1.2	AY525638.1	F: CGGCTTCGACTACAGCTTCT R: AGTGGGACACCACAGGGTAG
18S	AB778770.1	F: CAACGGATATCTCGGCTCTC R: TTGCGTTCAAAGACTCGATG

### Statistical Data Analysis

Analysis of variance (GLM; SAS Institute, 2008) was performed according to the experimental design. When no interaction between treatments occurred, treatment means were compared using Tukey’s honest significant difference (HSD_0.05_) at the 5% probability level. When an interaction between treatments occurred, *p*-values at the 5% probability level for differences of the LSMEANS (*p*diff) were used to separate interaction means.

Data on transporter expression were standardized to a mean of 0 and a standard deviation of 1. Canonical discriminant analysis (Klecka, 1980) (CDA; procedure CANDISC; SAS Institute, 2008) was run on standardized data to summarize the mean variation in transporter expression across all treatments.

## Results

### Root Mycorrhizal Colonization and Rhizoplane Colonization by Bacteria

Fertilization did not affect root colonization by AMF (**Table 2**). Soil inoculation with AMF (either alone or in combination with PGPR) markedly increased root colonization by AMF. No interaction among treatments on root AM colonization was observed; however, root AM colonization of treatments inoculated with PGPR alone was slightly higher in unfertilized than fertilized treatments (32.3 and 23.8%, respectively, *p*diff at LSMEANS = 0.0535).

**Table 2:** Aboveground biomass, N, and P contents of durum wheat (*Triticum durum*) at tillering and heading stages grown in the soil under unfertilized conditions or fertilized with an organic fertilized with low C:N ratio.

Treatments		Above ground biomass		Above ground N				Above ground P		Mycorrhizal infection	Rhizoplane colonization by bacteria
		Tillering	Heading	Tillering	Heading	Tillering	Heading	Tillering	Tillering	Tillering	Tillering
						
N application	Inoculation	(Mg ha^-1^)		(g N kg^-1^ DM)		(kg ha^-1^)		(g P kg^-1^ DM)	(kg ha^-1^)	(%)	Log_10_ number g^-1^ fresh root
Unfertilized	NAT	1.52	4.66	18.8	11.4	28.4	52.9	3.60	5.4	23.8	8.11
	+AMF	1.85	5.12	16.9	12.1	31.3	61.9	3.37	6.2	49.4	8.82
	+PGPR	1.51	5.08	17.6	11.4	26.7	58.0	3.38	5.0	32.3	9.18
	+AMF+PGPR	1.89	5.00	17.8	11.3	34.2	56.4	3.25	6.2	53.3	9.32
Fertilized	NAT	2.48	6.13	15.3	11.0	38.2	67.4	2.38	5.8	24.8	9.76
	+AMF	2.71	7.26	17.7	11.1	48.2	81.1	2.13	5.8	50.8	9.99
	+PGPR	3.00	6.82	19.8	11.7	58.2	79.9	2.53	7.4	23.8	10.09
	+AMF+PGPR	2.91	7.64	18.6	11.5	54.2	88.1	2.65	7.7	55.5	10.18
N application (F)	*P*-value	**0.002**	**<0.001**	0.880	0.328	**0.002**	**<0.001**	**0.014**	0.102	0.614	**0.024**
Inoculation (I)	*P*-value	**0.048**	**<0.001**	0.900	0.282	**0.011**	**<0.001**	0.335	**0.036**	**<0.001**	**0.026**
	HSD_(0.05)_	0.275	0.350	–	–	6.590	4.56	–	0.91	6.12	0.62
F × I	*P*-value	0.130	**0.010**	**0.029**	**0.038**	**0.018**	**0.005**	0.081	**0.020**	0.238	**0.042**

*Soil was left with the native microbial inoculum (NAT); inoculated with only arbuscular mycorrhizal fungi (AMF) spores; only plant growth-promoting rhizobacteria (PGPR), or both AMF+PGPR. Values in bold are significant at *P* ≤ 0.05. Tukey’s honest significant difference (HSD) at *P* = 0.05 is showed.*

Fertilization increased by 13.0% the Log_10_ number of bacteria on the rhizoplane. Under unfertilized conditions, inoculation with either AMF or PGPR increased Log_10_ number of bacteria on the rhizoplane, whereas under fertilized conditions, only PGPR (inoculated either alone or in combination with AMF) increased the density of bacteria on the rhizoplane (**Table 2**).

### Plant Growth

At tillering, both N fertilization and soil inoculation with plant growth-promoting microorganisms significantly affected the aboveground biomass of wheat. Compared to NAT, inoculation with both microrganisms (AMF or PGPR, or both) increased the aboveground biomass yield in both the fertilized and unfertilized treatments. The effects of soil inoculation on total N in the plant aboveground biomass varied according to the fertilizer treatment: in the unfertilized treatments, inoculation with AMF slightly, but not significantly, increased total N compared to NAT, whereas in the fertilized treatments, both PGPR and AMF, either singly, or co-inoculated, increased total N compared to NAT. However, in unfertilized plots, soil inoculation with plant growth-promoting microorganisms did not influence plant N concentrations (*p*diff at LSMEANS = 0.987), whereas in fertilized treatments, inoculation with PGPR significantly increased plant N concentration compared to NAT (*p*diff at LSMEANS = 0.012). Fertilization with an organic fertilizer strongly reduced the wheat P concentration (on average 3.40 and 2.42 mg P kg^-1^ biomass in unfertilized and fertilized plots, respectively). The effects of fertilization and soil inoculation with both microorganisms (AMF, PGPR, or both) on total aboveground P were very similar to those observed for total N.

At heading, soil inoculation with plant growth-promoting microorganisms always increased, compared to NAT, aboveground biomass and N accumulation in both unfertilized and fertilized treatments. For both of these traits, the greatest advantage was seen for AMF+PGPR, but only when the crop was fertilized. The effect of the inocula on the biomass N concentration at heading varied by fertilization treatment: in the unfertilized condition, soil inoculation with only AMF (+AMF) increased the N concentration compared to NAT, whereas in the fertilized condition, inoculation with PGPR (either alone or in combination with AMF) resulted in a higher N concentration than NAT.

### Gene Expression Analysis

#### Pi Transporters

**FIGURE 1:**
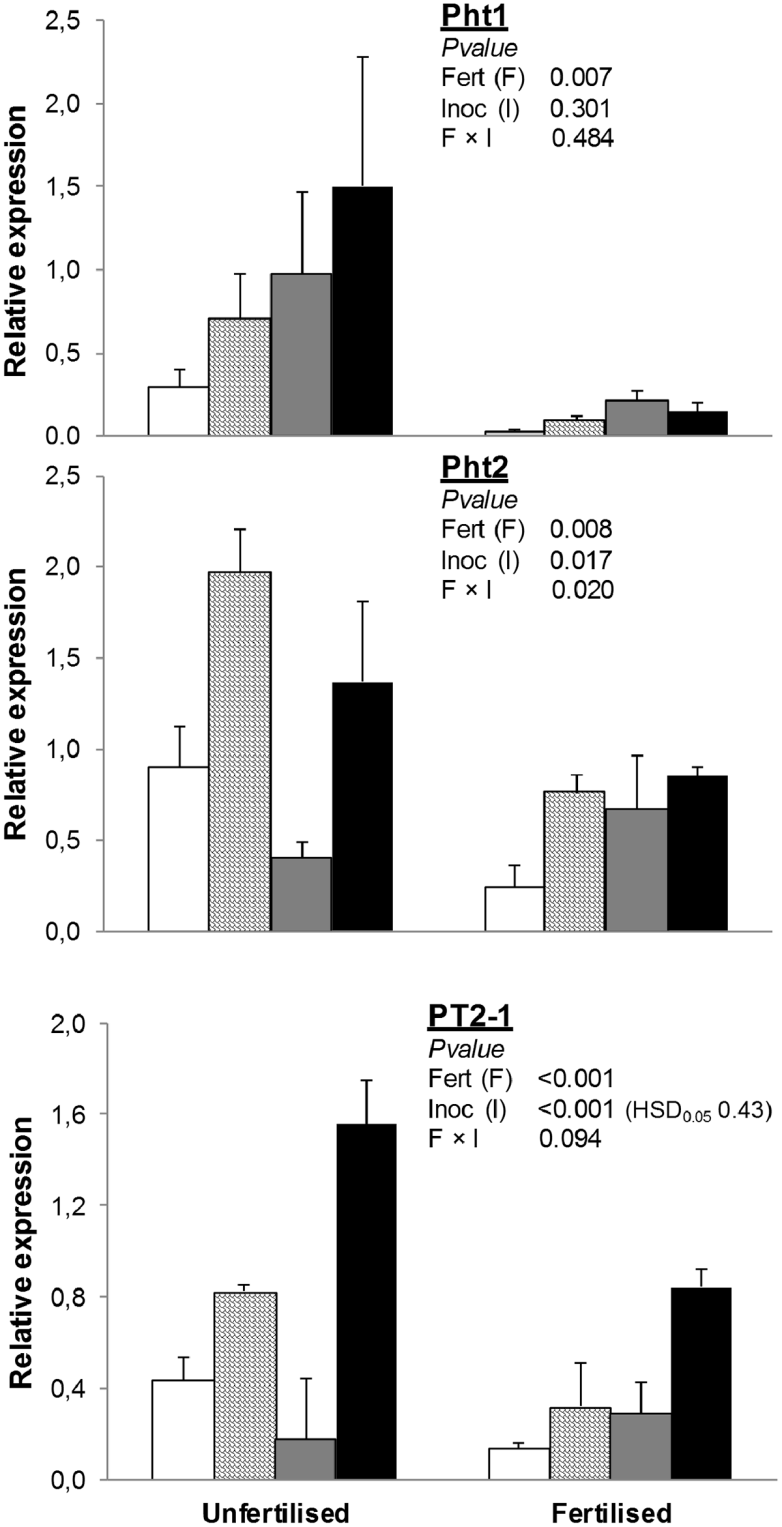
**Expression of phosphate transporter genes (*Pht1, Pht2*, and *PT2-1*) in *Triticum durum* root.** Plants were grown under the unfertilized conditions or fertilized with an organic fertilized with low C:N ratio. Soil was left with the native microbial inoculum (NAT, white bars); inoculated with only arbuscular mycorrhizal fungi spores (AMF, scaled bars); only plant growth-promoting rhizobacteria (PGPR, gray bars), or both AMF+PGPR (black bars). Means (*n* = 6) with standard errors, and analysis of variance results are shown. Fert is for fertilization treatment, Inoc for Inocula. Tukey’s honest significant difference (HSD_0.05_) of Inocula is shown when Inocula, but not Fertilizer × Inocula interaction, is significant.

Fertilization decreased the expression of *Pht1* by 86% and *PT2-1* by 49% (**Figure 1**). Inoculation with any or both of the plant growth-promoting microorganisms used in this study (AMF and/or PGPR) increased *PT2-1.* Under unfertilized conditions, inoculation with AMF significantly enhanced *Pht2* expression compared to NAT, whereas inoculation with PGPR downregulated it. Expression of *Pht2* in AMF+PGPR was similar to that observed in treatments inoculated with AMF alone. Under fertilized conditions, no differences were found in *Pht2* expression among inoculation treatments.

#### N Transporters

**FIGURE 2:**
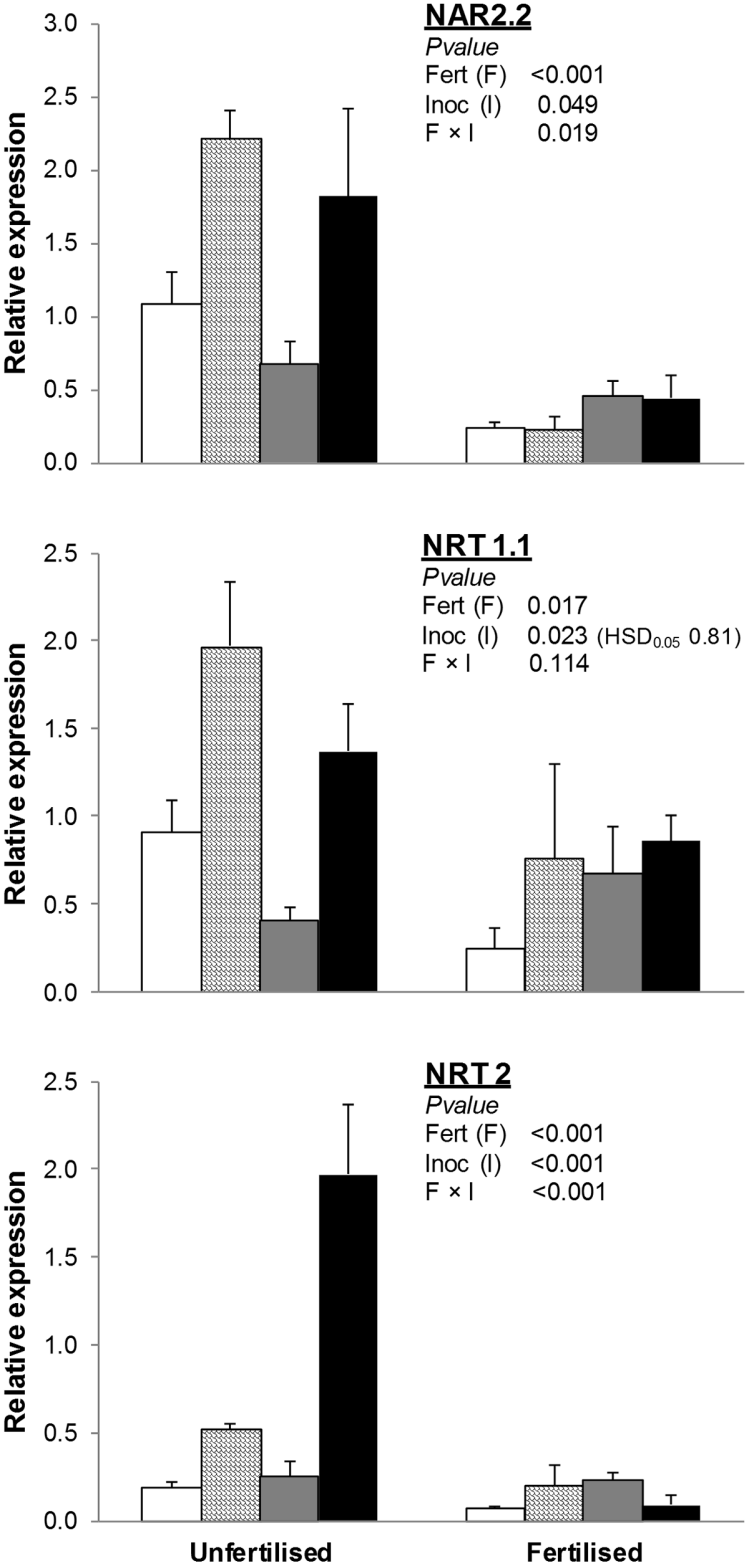
**Expression of nitrate transporter genes (*NAR2.2, NRT1.1, NRT2*) in *T. durum* root.** Plants were grown under the unfertilized conditions or fertilized with an organic fertilized with low C:N ratio. Soil was left with the NAT (white bars); inoculated with only AMF spores (scaled bars); only plant PGPR (gray bars), or both AMF+PGPR (black bars). Means (*n* = 6) with standard errors and analysis of variance results are shown. Fert is for fertilization treatment, Inoc for Inocula. Tukey’s HSD_0.05_ of Inocula is shown when Inocula, but not Fertilizer × Inocula interaction, is significant.

*NRT1.1* was significantly lower in fertilized than unfertilized treatments, whereas soil inoculation with AMF and AMF+PGPR strongly increased its expression compared to NAT and PGPR, respectively (**Figure 2**).

The effects of soil inoculation with plant growth-promoting microorganisms on the expression of *NRT2* and *NAR2.2* varied by fertilization treatment. In unfertilized conditions, differences among inocula were similar to those observed for *NRT1.1*. In fertilized treatments, no differences in the expression of *NRT2* and *NAR2.2* were observed among inoculation treatments.

**FIGURE 3:**
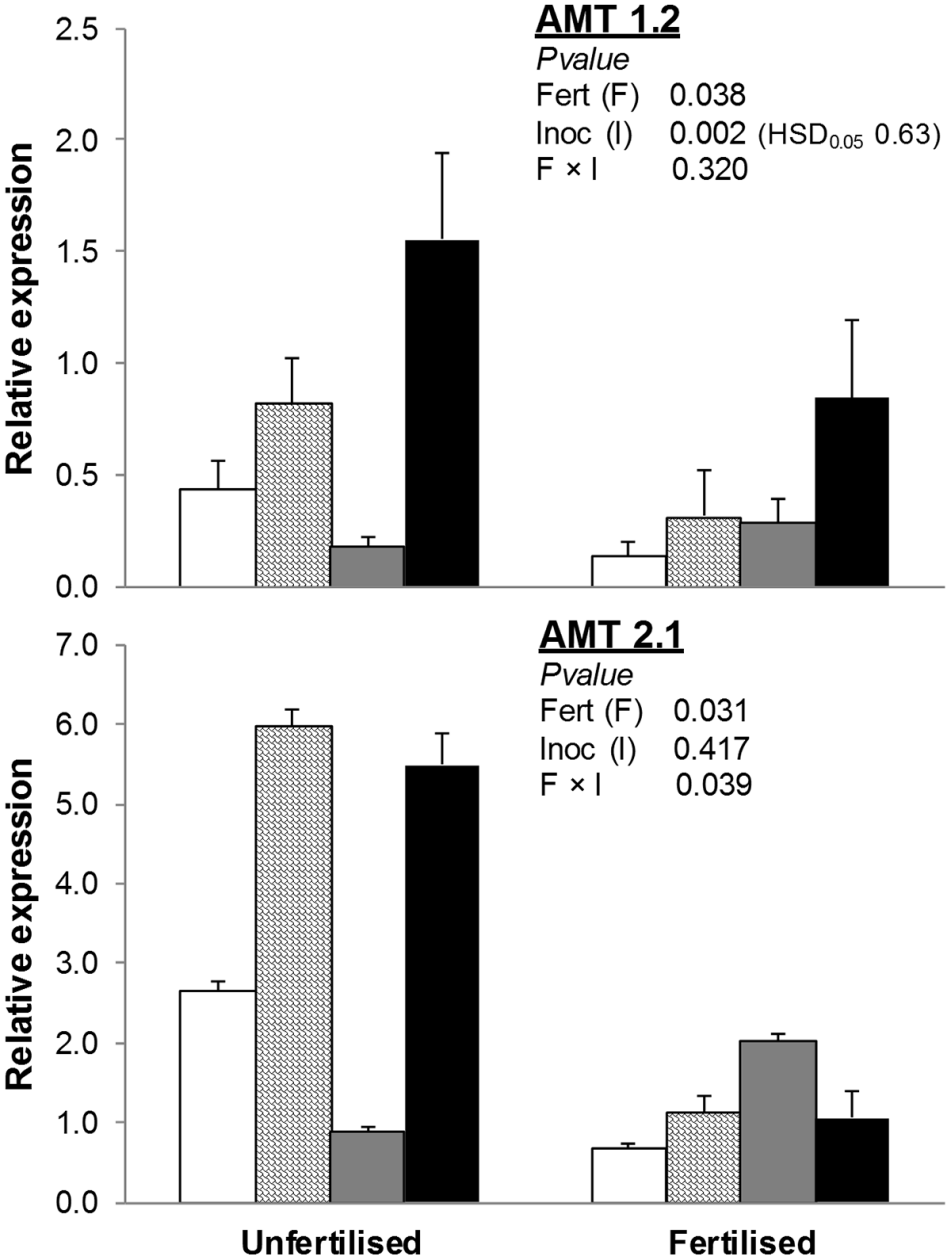
**Expression of ammonium transporter genes (*AMT1.2, AMT2.1*) in *T. durum* root.** Plants were grown under the unfertilized conditions or fertilized with an organic fertilized with low C:N ratio. Soil was left with the NAT (white bars); inoculated with only AMF spores (scaled bars); only plant PGPR (gray bars), or both AMF+PGPR (black bars). Means (*n* = 6) with standard errors and analysis of variance results are shown. Fert is for fertilization treatment, Inoc for Inocula. Tukey’s HSD_0.05_ of Inocula is shown when Inocula, but not Fertilizer × Inocula interaction, is significant.

The addition of an organic fertilizer to the soil also reduced both *AMT1.2* and *AMT2.1* (-47 and -67% compared to unfertilized treatments; **Figure 3**). Inoculation with AMF (alone or with PGPR) increased *AMT2.1* in unfertilized but not fertilized treatments. Finally, expression of *AMT1.2* was higher in AMF+PGPR than all other inoculation treatments (AMF or PGPR alone or NAT).

#### CDA

**FIGURE 4:**
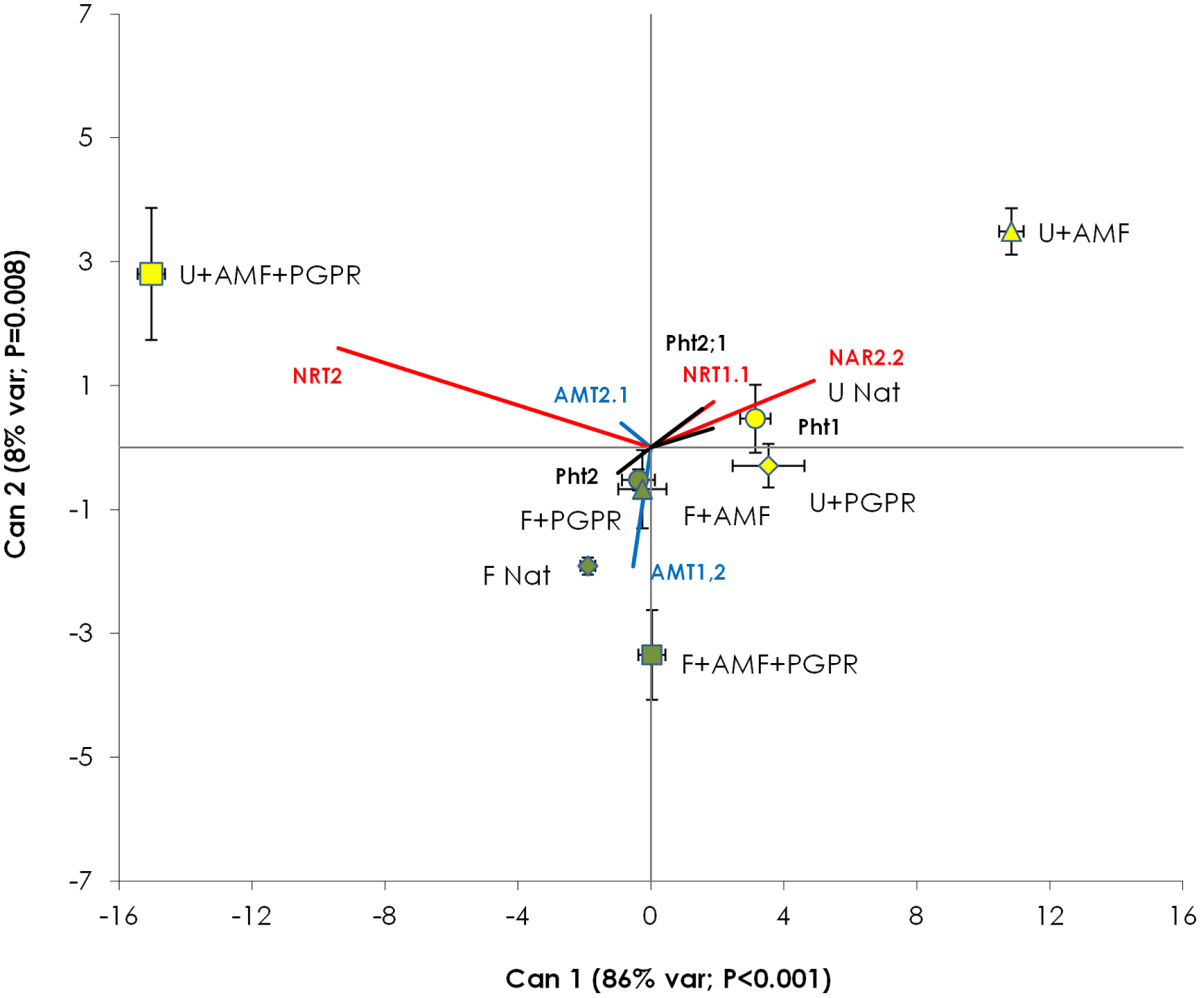
**Canonical discriminant analysis constructed with the gene expression data of *T. durum* root.** Percentage of total variance explained by each canonical axis is shown in parentheses. Percentages of variance explained by Can 3 to Can 7 were not significant (data not shown). Plants were grown under the unfertilized conditions (U, yellow symbols) or fertilized with an organic fertilized with low C:N ratio (F, green symbols). Soil was left with the NAT (circles); inoculated with only AMF spores (triangles); only plant PGPR (diamonds), or both AMF+PGPR (squares). Nitrate transporters vectors are shown in red, AMTs vector in blue and phosphorus transporters vector in black. Each symbol represents the treatment centroid within Can 1 and Can 2. Bars represent the standard error of the distribution of each treatment.

Canonical Variable (Can) 1 accounted for 86% of the total variance (*P* < 0.001) and Can 2 accounted for 8% of the total variance (*P* = 0.008). Can 1 mostly varied according to *NRT2* (score = -9.41) and *NAR2.2* (score = +4.91), whereas Can 2 was mostly influenced by *AMT1.2, NRT2*, and NAR2.2 (scores = -1.92, 1.60, and 1.08, respectively). Can 1 did not discriminate among fertilized and unfertilized treatments. CDA (**Figure 4**) clearly differentiated samples from AMF-inoculated, unfertilized plots from all other treatments (with *P* > Mahalanobis distance always less than 0.003), whereas the other treatments (i.e., unfertilized NAT and unfertilized PGPR and all the fertilized treatments) were grouped together.

## Discussion

### Root Mycorrhizal Colonization, Rhizoplane Colonization by Bacteria and Plant Growth

Soil inoculation with plant growth promoting microbes, such as PGPR and AMF, is a promising tool of integrated management systems, and many efforts have been made to increase the efficiency of plants’ use of nutrients (from either soil or fertilizers) through microbial technology and the sustainability of the cropping systems. In the present work, the effects of soil inoculation with AMF and PGPR efficient at promoting plant growth were studied in durum wheat grown in an area characterized by poor N availability and soil organic carbon content. We found that inoculation with AMF, PGPR, or both increased rhizoplane colonization by bacteria, which is an important indicators of soil quality (Schloter et al., 2003). And this occurred especially under unfertilized conditions. Root colonization by the natural AM consortium (NAT) was lower than that observed by other authors in the same species (Gao et al., 2010). Soil inoculation with AMF spores markedly increased root AM colonization and rhizoplane colonization by bacteria. Teng et al. (2013) observed that natural AM colonization decreased with increasing P supply to the soil. In our experiment, both available and total soil P content were very high (92 ppm and 1370 ppm, respectively), and this, along with the huge amount of P fertilizer usually applied in the area, may have contributed to the selection of less beneficial AM species (Ehinger et al., 2009). Nonetheless, the increase in root AM colonization after soil inoculation with AMF, as also observed elsewhere (Al-Karaki et al., 2004), suggests that other factors could be detrimental to the colonization process by the NAT, including the effect of soil organic matter content on AMF growth (Kohler et al., 2015) or continuous soil plowing. Indeed, soil inversion plowing disrupts the AM hyphal net, which usually represents the most important source of inoculum, and displaces spores in the deep soil layer, where root growth is delayed in the growing season (Kabir, 2005). In addition, plowing may select for sporulating AM fungal genotypes, which invest more resources in sporulation rather than symbiotic activity (Jansa et al., 2003).

Soil inoculation with AMF increased plant growth irrespective of fertilization, and this resulted in a higher aboveground biomass yield and N and P uptake in comparison with uninoculated treatments. These results agree with those obtained in other experiments carried out in both field and controlled (pot) conditions (Adesemoye et al., 2008; Berta et al., 2014; Saia et al., 2014a,b). As observed by Baris et al. (2014) in spring wheat and barley, in our experiment soil inoculation with PGPR increased the aboveground biomass and N and P uptake in comparison with uninoculated treatments, but only when organic fertilizer was applied. Several studies have shown that the advantages of PGPR can be attributed, among other factors, to a more rapid breakdown of organic matter, which enhances the availability of nutrients for plants (Yildirim et al., 2011). The delay in the benefit of PGPR compared to AMF in terms of N uptake may be due to the time required by PGPR for the mineralization processes, the amount and quality of wheat root exudates and root biomass, or the reduced availability of carbon for bacteria (Hu et al., 2009), as the effect of PGPR at tillering was evident only in the organic fertilized treatments.

### Pi and N Transporters

Besides the adaptive strategies adopted by plants to increase P absorption, such as secreting phosphatases, organic acids, and protons (Dunlop and Gardiner, 1993) or enhancing root growth and/or modifying root morphology (Bates and Lynch, 1996), positive correlations between AMF symbiosis formation and shoot biomass, P uptake, and total P content have been reported (Avio et al., 2006). In the present experiment, fertilization reduced all P transporters, although the effects of the inocula varied depending on the fertilization treatment: under uninoculated treatments, inoculation with AMF increased the expression of both *Pht2.1* and *Pht2*, the latter of which was also increased by PGPR in fertilized treatments. The effects of fertilization on total P uptake, but not those of soil inoculation with both microorganisms, complied with the expression of P transporters: indeed, total P uptake increased after fertilization, which suggests that fertilization resulted in an increase in the available P fraction in soil, and this may have been due to soil acidification by the soil bacteria when mineralizing the organic fertilizer (Bertrand et al., 2007). Because inoculation with PGPR increased plant growth and total P uptake in fertilized treatments, we should have observed a reduction in the expression of P transporters compared to uninoculated treatments (NAT). Nonetheless, P transporters of wheat in plots inoculated with PGPR were higher than NAT. This implies that PGPR can stimulate P uptake through a direct effect on plant metabolism (Richardson et al., 2009). The role and importance of AM and/or PGPR in plant N nutrition is uncertain, and it is not clear under which conditions AM is beneficial for N uptake. Consistent with their specific function, many members of the NRT1 and NRT2 families are involved in the uptake of nitrates from the soil into the root and their translocation to the shoots (Fan et al., 2009). In particular, the dual-affinity *NRT1.1* transporter is triggered by a wide range of soil nitrate concentrations, and its switching from LATS to HATS functionality is determined by a phosphorylation at Thr101 (Ho et al., 2009). Here, *NRT1.1* transcript abundance was influenced by both inoculation with AMF and fertilization, confirming its dual-affinity function.

In contrast, the *NRT2.1* member of the NRT2 family encodes a HATS of nitrate uptake (Huang et al., 1999). The expression of *NRT2* from *Triticum aestivum* with high homology with *AtNRT2.1* under unfertilized conditions was significantly induced, in *T. durum*, by inoculation with AMF+PGPR compared to the uninoculated control. A less significant increase in *NRT2* expression was also induced by inoculation with AMF. As expected, a downregulation in *NRT2* was observed when N80 organic fertilizer was supplied. The upregulation of both *NRT1.1* and *NRT2* by inoculation with AMF and AMF+PGPR is consistent with the increased aboveground N compared to NAT. These results suggest that in unfertilized plots, the increased N accumulation in the AMF-inoculated plant biomass is mediated by the upregulation by AMF of nitrate transporter genes. In terms of the classification of nitrate transporters as constitutive, repressible, and inducible (Wang et al., 2003), our results show that both *NRT1.1* and *NRT2* seem to be nitrate-repressible genes, although *NRT2* was more downregulated than *NRT1.1* under fertilized conditions, according to its nitrate dual affinity (Ho et al., 2009).

Moreover, NRT2.1 may interact with an NAR2-type protein for a functional HATS based on the essential role of NAR2.1 in *Arabidopsis* (Orsel et al., 2006). Under unfertilized conditions, *NAR2.2* was significantly induced by inoculation with AMF and AMF+PGPR compared to the uninoculated control. Thus, like *NRT2*, its HATS partner *NAR2.2* was highly inhibited by organic N fertilization. Given *NRT2/NAR2.1* expression and the relative protein interaction, first reported in *Arabidopsis* (Orsel et al., 2006), here we have shown an AMF can have a role in mediating the expression of *NRT2/NAR2.1* in durum wheat. In particular, the two genes seemed to be upregulated by inoculation with AMF and AMF+PGPR, and this was probably mediated by a reduced availability of ammonium in AMF and AMF+PGPR than NAT (Saia et al., 2015). In particular, the presence of AMF highly upregulated *NAR2.2.* In contrast, *NRT2/NAR2.2* was strongly downregulated by N fertilization per HATS functionality but probably also through the increase in the availability of NH_4_^+^ in fertilized soils.

Such as observed in nitrate transporters, we also found that in unfertilized conditions, the expression of the HATS *AMT1.2* was significantly increased when wheat was inoculated with AMF+PGPR compared to NAT; a positive, though not significant, effect was also observed with inoculation with AMF.

These results do not completely agree with the AMT2 gene family low-affinity function, but previous findings showed that the regulation of both AMT family genes is controlled by a complex network of different N forms and concentrations (Glass et al., 2002).

## Conclusion

In conclusion, the results of the present study showed that soil inoculation with AMF increased plant growth and N uptake of durum wheat compared to the uninoculated control irrespective of fertilization. CDA suggested that the effects of the inoculation with AMF on the expression of P and N transporters in the plant root were evident only in unfertilized condition. Soil inoculation with PGPR benefitted plant growth and nutrient uptake only when organic fertilizer was added.

Agronomic benefits from the soil inoculation with beneficial microbes could depend on the availability of nutrient for the microbe: AMF, which receive photosynthates only from the host plant, in our experiment benefitted the crop under both fertilized and unfertilized conditions, whereas PGPR, which can also take C from the soil, benefitted the crop only in plots where the organic fertilizer was added.

These results indicate soil inoculation with AMF and PGPR (alone or in combination) is a valuable option for farmers to improve nutrient uptake and the sustainability of the agro-ecosystem. Further studies are needed to evaluate the benefit of the soil inoculation with efficient consortia of both AMF and PGPR at varying the doses and characteristics of the fertilizers applied.

## Author Contributions

SS, DG, and FM conceived and designed the experiments. Experimental work was performed by SS, VR, FM. Data were analyzed and discussed by SS, PR, ASF, MRA, FS, FM, and DG. AF and DG contributed to reagents and materials. Manuscript was written by FM, SS, PR, MRA, ASF, and DG.

## Conflict of Interest Statement

The authors declare that the research was conducted in the absence of any commercial or financial relationships that could be construed as a potential conflict of interest.
